# Predictive Processing in Autism Spectrum Disorder: The Atypical Iterative Prior Updating Account

**DOI:** 10.1016/j.bpsgos.2025.100468

**Published:** 2025-02-17

**Authors:** Zhuanghua Shi, Fredrik Allenmark, Laura A. Theisinger, Rasmus L. Pistorius, Stefan Glasauer, Hermann J. Müller, Christine M. Falter-Wagner

**Affiliations:** a Department of Psychology, LMU Munich, Munich, Germany; b Department of Psychiatry and Psychotherapy, LMU University Hospital, LMU Munich, Munich, Germany; c Computational Neuroscience, Brandenburg University of Technology Cottbus-Senftenberg, Cottbus, Germany; d Faculty of Health Sciences Brandenburg, Brandenburg University of Technology Cottbus-Senftenberg, Cottbus, Germany

**Keywords:** Autism, Bayesian modeling, Iterative updating, Perception, Prior belief

## Abstract

**Background:**

The nature of predictive-processing differences between individuals with autism spectrum disorder (ASD) and typically developing (TD) individuals remains contested. Some studies have reported impaired predictive processing in ASD, while others have suggested intact but atypical learning dynamics.

**Methods:**

We investigated duration reproduction tasks under high- and low-volatility settings to examine the updating dynamics of prior beliefs and sensory estimate updating in individuals with ASD (*n* = 32) and TD counterparts (*n* = 32). Using a two-state Bayesian model, we analyzed how the participants updated their prior beliefs and perceptual estimates and how these updates affected their behavior over time.

**Results:**

Individuals with ASD integrated prior knowledge similarly to TD control participants for perceptual estimates. However, they relied more heavily on sensory input for iteratively updating their prior beliefs, perceiving events as less interconnected. This heightened reliance on sensory inputs led to the initial underweighting of priors in perceptual estimates, resulting in a weaker central tendency early in sessions. Over time, ASD participants adapted, reaching integration weights comparable to those of TD control participants by the end of the session. These findings suggest that predictive processing in ASD is characterized by distinct updating dynamics, not an inability to form or use prior effectively.

**Conclusions:**

Our study highlights a unique interplay between sensory inputs and prior beliefs in ASD, where greater reliance on sensory inputs during prior updating influences adaptation speed and intertrial dynamics. This process clarifies inconsistencies in the literature and underscores the role of interactive updating in predictive processing differences between individuals with ASD and TD individuals.

## Plain Language Summary

This study explores how individuals with autism spectrum disorder (ASD) process sensory information and update their beliefs during predictive tasks. Using a duration reproduction task, researchers found that while people with ASD can use prior knowledge like typically developing (TD) individuals, they rely more on sensory input for belief updates. This results in slower adaptation and a weaker initial central tendency. These findings highlight unique updating dynamics in ASD, resolving inconsistencies in past research and emphasizing differences in how sensory inputs and prior beliefs interact in predictive processing.

Autism spectrum disorder (ASD) presents challenges in social interaction, communication, and repetitive behaviors, often accompanied by heightened sensory sensitivity and adaptation difficulties (1, 2, 3). Predictive processing and Bayesian inference offer promising frameworks for understanding the autistic cognitive profile, highlighting a distinct approach to processing sensory information and prior knowledge in ASD (4, 5, 6, 7, 8, 9, 10, 11, 12). According to Bayesian inference, perception and action result from continuously refining predictions and minimizing prediction errors by integrating sensory input with prior knowledge, weighted by their associated uncertainties. While theories agree that predictive processing differs in ASD, they disagree on the exact mechanisms causing these differences, leading to two broad model categories.

The attenuated-prior model argues that individuals with ASD have weaker priors, resulting in a reduced reliance on the priors in making predictions (4,13,14). Conversely, according to the “heightened and inflexible precision of prediction errors in autism” theory, sensory atypicality in ASD is attributable to an overemphasis on prediction errors (6). Lawson *et al.* (5) offered a similar view, highlighting an impaired ability in ASD to downweight sensory input, skewing the balance toward sensory input over prior beliefs in perceptual decisions. Despite different explanations, both types of models agree that individuals with ASD have distinct predictive processing (7) and tend to prioritize sensory information.

Contrary to the view that individuals with ASD form compromised prior beliefs, many types of prior—particularly those deriving from experience or top-down knowledge—actually remain intact (15). For example, individuals with ASD can accurately form one-shot priors, such as recognizing a Dalmatian dog camouflaged by black patches (16), and they show typical performance on tasks related to the processing of gaze direction (17,18), statistical learning of likely distractor locations (19), and using external reference coordinates in tactile spatial tasks (20). These observations have led Palmer *et al.* (8) to challenge the adequacy of simplistic Bayesian models that only account for the integration of sensory input with a single prior. Instead, they proposed that the peculiarities in ASD may stem from altered expectations, in hierarchical inference, about the likelihood (or uncertainty) of changes occurring in the hidden states governing the stimulus environment (referred to as meta-volatility). To explore this, Lawson *et al.* (10) devised a discrimination task in which participants responded to probabilistic cues (either a high or low tone) predicting an upcoming stimulus. They found that individuals with ASD exhibited less variation in response times and pupil dilation when faced with expected versus unexpected outcomes. Computational modeling indicated that these individuals exhibited diminished surprise about unexpected outcomes and heightened sensitivity to uncertainty, leading them to overestimate the volatility of their environment. On the other hand, Manning *et al.* (21) found no such group difference in a reward-probability learning task (learning the likelihood of a chest containing a reward) involving children with ASD and matched TD counterparts. Both groups adapted appropriately to changing volatility, suggesting that predictive mechanisms impacting perception in ASD may not extend to learning tasks. This null finding is consistent with recent indications that individuals with ASD do not show a generalized learning deficit but rather atypical responding to short-term prediction errors (19) or atypical updating of social expectations, such as other people’s intentions (22).

Notably, many relevant ASD studies have focused on interpreting averaged outcomes as representing the percept, overlooking a potentially important aspect: that of short-term, iterative, and dynamic updating of priors from one trial to the next. Recent reports indicate that individuals with ASD may be less influenced by preceding events (23) and may adapt more slowly to changes (24) than their matched peers. Thus, we hypothesized that distinctive dynamic updating patterns might not necessarily compromise long-term prior formation but could impact average outcomes across different timescales. Extended learning, rather than short-period adaptation, may enable individuals with ASD to develop priors that are more similar to those of their TD counterparts. Using dynamic, iterative Bayesian updating models, we aimed to resolve discrepancies in the literature and enhance our understanding of the mechanisms underlying atypical responses in ASD.

Accordingly, we used a duration-reproduction task with varied sequential volatility (25) to differentiate between short-term-prior and perceptual-estimate updating and long-term integration. The reproduction task is widely recognized for its effectiveness in assessing the integration of sensory measurements with priors, thus facilitating the assessment of integration weights within a Bayesian framework (14,26,27). This paradigm also allows direct comparisons with previous work in ASD research, such as Karaminis *et al.* (14). Furthermore, the reproduction task supports the application of dynamic-updating models (25,28,29), enabling the analysis of rapid, short-term updates alongside slow, long-term adjustments (28), as well as the impact of trial-to-trial variability or volatility (29). Over 2 sessions, we kept the same set of durations while varying their sequential presentation volatility, thereby isolating the effects of short-term trialwise perceptual-estimate updating (local volatility) from iterative updating of the long-term prior. Crucially, we used iterative 2-state Bayesian modeling (29) to track both the integration of sensory inputs with priors and the dynamic updating of these priors, clearly delineating them from the influences of perceptual estimate updating.

## Methods and Materials

### Participants

Thirty-two individuals (13 female, aged between 18 and 67 years; mean [SD] = 32.0 [12.3] years) with a confirmed ASD diagnosis (F84.0 or F84.5) according to ICD-10 (30) were recruited from the database and network partners of the Outpatient Clinic for ASD at the Department of Psychiatry, LMU Munich, Germany. A total of 32 TD control participants (13 female, aged between 18 and 70 years; mean [SD] = 31.6 [13.6]) with no reported history of mental illnesses or neurological deficits were recruited via local advertising. The groups were matched pairwise using a measure of crystalline intelligence, the Mehrfachwahl-Wortschatz-Intelligenztest [MWT-B; multiple choice vocabulary test (31)]. The groups were comparable in terms of IQ and age, but differed significantly on the autism spectrum quotient (AQ) (32), empathy quotient (EQ) (33), systemizing quotient (SQ) (34), and Beck Depression Inventory (BDI) scores (35), for details, see Table S1.

A typical duration reproduction study (29) showed that 14 participants yielded significant central tendency and sequential dependence with a large effect size (Cohen’s *d* > 1.74), and a prior study (10) on volatility also revealed a large effect size (*d* = 1.024) between the ASD and TD groups. With a similar effect size, power of 0.8, and alpha of 0.05, the required sample size was estimated to be 26. To be cautious, we increased the sample size to 32. All participants provided written informed consent prior to the experiment and received compensation of €10/hour for their participation. The study was approved by the Ethics Board of the LMU Munich Faculty of Pedagogics and Psychology, Germany.

### Design and Procedure

The experiment was conducted in a sound-attenuated and moderately lit experimental cabin. A yellow disk patch (diameter: 4.7° of visual angle; luminance: 21.7 cd/m^2^) was used for delivering (stimulus) durations on a 21-inch LACIE CRT monitor (refresh rate: 85 Hz). The experimental code was developed using the MATLAB PsychToolbox (36).

**Figure 1:**
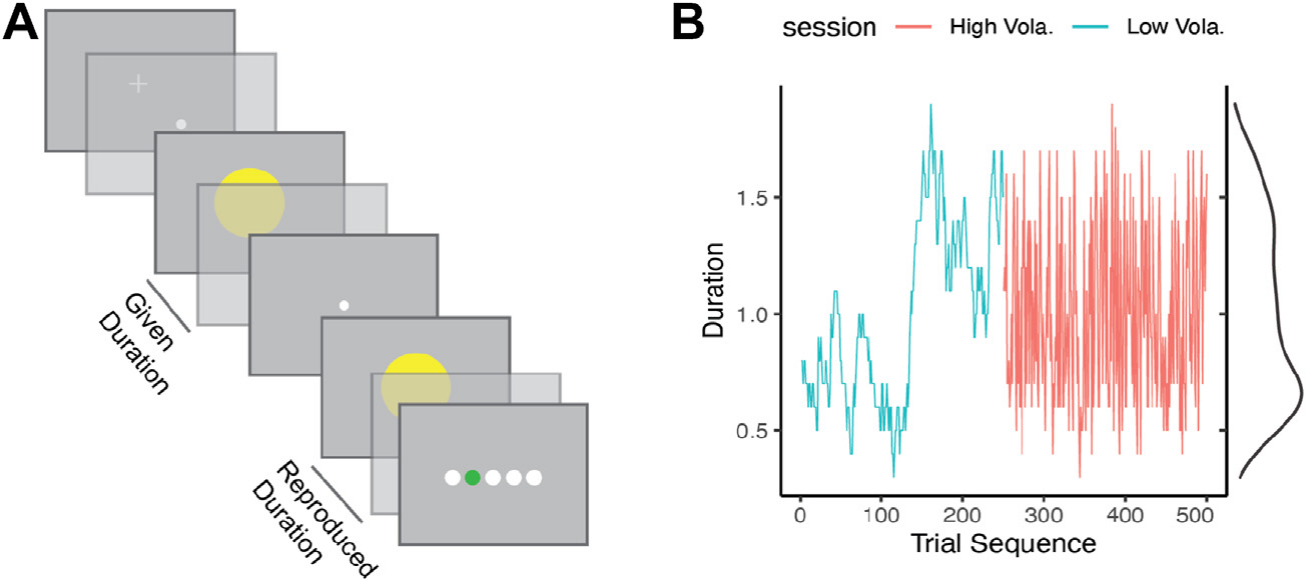
**(A)** Schematic illustration of a trial sequence used in the duration-reproduction task. **(B)** Example duration (trial) sequences in 2 consecutively performed duration-volatility sessions. The session depicted on the left (in cyan) represents a low-volatility sequence (Low Vola.), and the session depicted on the right (in red) represents a high-volatility sequence (High Vola.). Both sessions presented the exact same durations (i.e., the same density function depicted on the right), differing only in their presentation orders.

We adopted the duration-reproduction paradigm (37) (Figure 1A). Each trial started with a central fixation cross (0.75°) presented for 500 ms, followed by a central white dot (0.2°), signaling participants to press and hold either mouse button to initiate the duration-encoding phase. Upon the press, a yellow disk appeared on the center of the screen for a random duration (100–1900 ms; see details below). Participants released the button when the disk disappeared. A 500-ms blank screen separated the encoding and reproduction phases. During the reproduction phase, a white dot appeared, informing participants to press and hold the mouse button to match the observed duration, during which the yellow disk reappeared and disappeared upon button release. Subsequently, feedback display was presented for 500 ms, showing 5 horizontal disks representing reproduction error ranges: <−30%, −30% to −5%, −5% to 5%, 5% to –30%, and >30%. One of the disks changed color—green for minor errors (3 central disks) or red for large errors (2 outer disks)—to indicate the accuracy of the response. Red highlighted a significant bias, which participants were instructed to avoid.

The experiment consisted of 2 sessions, each comprising 10 blocks of 25 trials (250 trials total). One session featured a low-volatility sequence of stimulus durations, and the other featured a high-volatility sequence. Critically, both conditions consisted of the same set of stimulus durations and an equal number of duration repetitions; they differed only in terms of the presentation order. First, individually for each participant (i.e., matched pair of participants), a low-volatility sequence was generated via a random-walk process starting from a randomly selected duration. Each subsequent duration was derived by adding a slight random fluctuation, sampled from a normal distribution, to the previous duration. To ensure that all values remained within the 100- to 1900-ms range, durations exceeding these bounds were shifted and scaled back and rounded to the nearest 100 ms, allowing for multiple repetitions of each duration. This procedure resulted in a low-volatility sequence characterized by mild fluctuations across trials. The high-volatility sequence was created by randomly shuffling the low-volatility sequence, producing greater trial-to-trial variation. Figure 1B illustrates typical low- and high-volatility sequences across 2 sessions. Both sequences were generated before the experiment, and the session order was counterbalanced across participants. The 2 sessions were conducted back-to-back, with participants taking a self-paced break outside the cabin between sessions.

Importantly, we administered identical sequences to (age- and IQ-) matched participants in the ASD and TD groups, thereby ensuring that any differential effects observed would be attributable to differences between the 2 groups.

Prior to the experiment, participants received comprehensive written and verbal instructions, and they underwent a pre-experimental training block of at least 10 trials to ensure that they understood the instructions. Subsequently, the experiment was conducted, followed by filling out of the questionnaires and debriefing of the task. The debriefing revealed that participants did not notice any difference in the randomization (i.e., sequential duration volatility) regimens between the 2 sessions.

## Results

### The Central-Tendency Effect and Impact of Volatility

**Figure 2:**
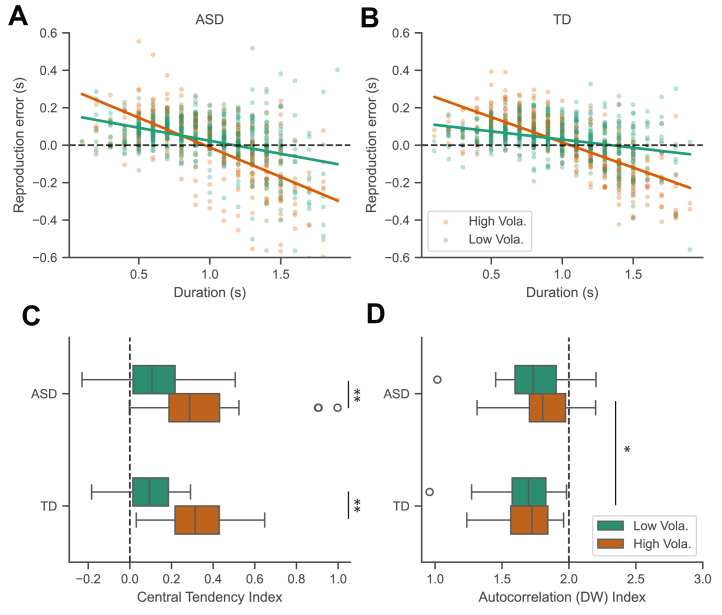
Average reproduction error as a function of the (to-be-reproduced) stimulus duration for the autism spectrum disorder (ASD) **(A)** and typically developing (TD) **(B)** groups (each group *n* = 32), separately for the high- (red) and low- (green) volatility sessions. Each individual dot represents a single participant’s mean reproduction error for that given duration. Dots above the horizontal dashed line indicate overestimations, and dots below the line indicate underestimations. Solid lines indicate the fitted trend of the reproduction errors. Notably, the steepness of the fitted trends is higher in the high- than the low-volatility session, indicative of stronger central-tendency biases during high-volatility conditions. **(C)** Boxplots of the central-tendency index (CTI) for the ASD and TD groups, separately for the high- and low-volatility sessions. A CTI of 0 implies a lack of a central-tendency effect, whereas a CTI of 1 indicates a pronounced central-tendency effect. The left and right boundaries of the box denote the interquartile range (IQR), from the 25th to the 75th percentile, while the whiskers indicate 1.5 times the IQR. Three outliers were observed in the ASD group during the high-volatility session. **(D)** The boxplots of the average Durbin-Watson (DW) autocorrelation indices, separately for the high- and low-volatility sessions, for both the ASD and TD groups. The dashed line, representing a DW value of 2.0, signifies the absence of autocorrelation, while a DW value <2 indicates a positive autocorrelation. ∗*p* < .05, ∗∗*p* < .01.

To assess the central tendency effect, we preprocessed reproduced durations by removing outliers—values outside (duration/3, 3 × duration)—which accounted for only 0.58% of trials. Given the linear nature of the central-tendency effect (25,38), we used the absolute value of the regression slope to estimate this effect, termed the central-tendency index (CTI). A CTI of 0 indicates no bias, while 1 reflects a pronounced central tendency. Both the ASD and TD groups exhibited a linear decrease in reproduction error with increasing duration (Figure 2). The high-volatility session showed a steeper linear trend, indicating a stronger central-tendency bias.

Figure 2C presents boxplots of the CTIs by volatility and group. A volatility-by-group mixed-design analysis of variance (ANOVA) on the CTIs yielded a robust main effect of volatility, *F*_1,62_ = 80.11, *p* < .001, η_p_^2^ = 0.56, with no significant effects for group or the volatility × group interaction (*F*s < 0.23, *p*s > .63).

In the ASD group, 3 outlier participants exhibited a substantial central-tendency effect (CTI > 0.9), reproducing similar durations across all tested durations in the high-volatility session (Supplement 2). Excluding these 3 outliers and their matched controls did not change statistical outcomes (volatility: *F*_1,56_ = 81.37, *p* < .001, η_p_^2^ = 0.59; others: *F*s < 3.324, *p* > .074).

Moreover, reproduction errors skewed toward overestimation. A volatility-by-group mixed-design ANOVA on errors revealed a significant main effect of volatility, *F*_1,62_ = 5.57, *p* = .021, η_p_^2^ = 0.08, with greater overestimation in low-volatility (39 ms) than high-volatility (22 ms) sessions. Neither group nor the group-by-volatility interaction was significant, *F*s < 0.698, *p*s > .4, η_p_^2^s < 0.01.

### Autocorrelation Reveals a Reduction of Positive Correlation in ASD

If reproduction errors were solely driven by the central tendency effect, the residuals from the regression should represent independent random errors. However, the residual analysis revealed a marked differential autocorrelation, showing a dependency of the reproduction error from the preceding trial (*n* − 1). Figure 2D shows average Durbin-Watson (DW) statistics for the 2 groups across sessions. The DW statistics, which ranges from 0 to 4, measures autocorrelation between trials *n* and *n* − 1. A DW value of 2 suggests no autocorrelation, while values < 2 indicate positive autocorrelation.

The DW scores for both groups were significantly below 2 (*t*s < −7.9, *p*s < .001), confirming positive autocorrelation. A further mixed-design ANOVA on DW revealed a significant group difference, *F*_1,62_ = 4.50, *p* = .038, η_p_^2^ = 0.07 (excluding outliers: *F*_1,56_ = 8.36, *p* = .005), showing that autocorrelation was significantly less in the ASD group than in the TD group. These findings indicate that central tendency alone did not fully capture intergroup differences, particularly regarding intertrial dependencies. However, the main effect of volatility and the volatility × group interaction failed to reach significance (*F*s < 2.19, *p*s > .145).

### Distinct Prior Updating Revealed by Two-State Iterative Model

To more closely model intertrial dependence, we used the two-state model (29), a hybrid Bayesian iterative-updating framework that accounts for both long-term central-tendency effects and short-term intertrial sequential dependencies. This model posits that an individual’s internal prior belief, represented by a prior distribution, evolves iteratively by incorporating new sensory information, thereby forming a revised belief, the posterior distribution. The posterior is used to compute an estimate, often with a certain cost function like the maximum a-posteriori.

**Figure 3:**
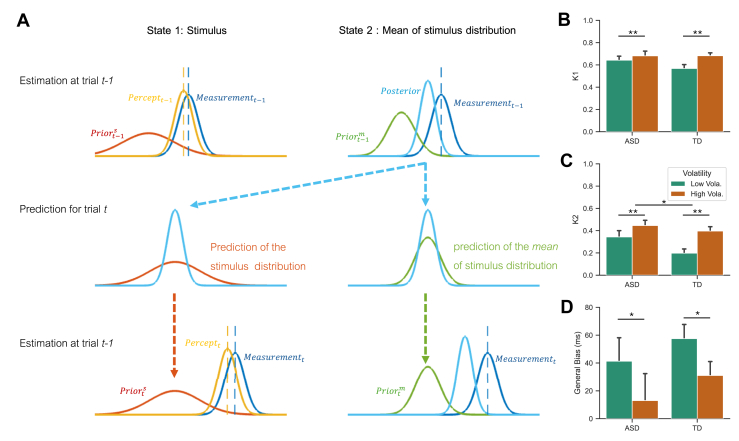
**(A)** Two-state iterative Bayesian updating model. The left panels illustrate the iterative updates of the perceptual estimate of the stimulus (state 1), while the right panels depict the iterative updates of the mean of stimulus distribution (state 2). On trial *t* − 1 (top row), the percept (yellow) is computed by multiplying the prior of the stimulus (red) and the sensory likelihood (dark blue), which corresponds to Eq. 4 in Supplement 3. The posterior distribution (light blue) of the stimulus mean is derived by combining the prior of the mean distribution (green) with the sensory likelihood (dark blue), which corresponds to Eq. 5 in Supplement 3. After trial *t* − 1 (middle row), the stimulus mean distribution (light blue) for trial *t* is updated from the posterior distribution (indicated by the dashed blue arrow). The stimulus prior (red) for trial *t* is then formulated for the prediction. Additionally, the prediction of the mean of stimulus distribution (green) for trial *t* is computed from the posterior distribution (light blue). On trial *t* (bottom row), these processes are repeated as in trial *t* − 1. **(B)** Estimated Kalman gain $K_{1}$ for the state of duration prediction; **(C)** estimated Kalman gain $K_{2}$ for the updating of the prior belief; **(D)** general overestimation (ms), separately for 2 groups (autism spectrum disorder [ASD] vs. typically developing [TD]) and volatility sessions (low vs. high volatility). Each group *n* = 32. Error bars denote 1 standard error. ∗*p* < .05, ∗∗*p* < .01.

Unlike traditional Bayesian models with fixed priors, the two-state model updates the mean of the prior distribution dynamically through a Kalman filter process (Figure 3A) (29). Each new sensory information is integrated with two Kalman gains (weights): $K_{1}$, influencing sensory estimation (state 1), and $K_{2}$, affecting updates to the mean prior (state 2). The 2 Kalman gains ($K_{1}$ and $K_{2}$) are latent variables that capture how estimates and beliefs adjust over time. Specifically, $K_{1}$ is responsible for the central tendency effect, while intertrial sequential bias is shaped by $K_{2}\left(1-K_{1}\right)$ in a steady state (see analytical proof and simulations in Supplement 3). To reflect a general bias, we included a constant bias parameter *c* in the state 1 updating.

The two-state model outperformed the standard linear regression (reported in the previous subsection), reducing the Akaike information criterion (AIC) by 10.75, which indicates a markedly better fit to the behavioral data. Additionally, we compared the two-state model to the dynamic-updating IRM (39). The IRM assumes that an internal reference is updated with the sensory input trial by trial and used for reproduction. Even though updating weight could reflect changes in environmental volatility and differences between groups, none of the factors became significant (Supplement 4). The model comparison revealed that our two-state model captured the behavioral findings clearly better than the IRM (average reduction of AIC: 11.25). Because the IRM can be interpreted as a steady version of the two-state model with one of the parameters fixed at zero, the model comparison strongly suggests that all model parameters are crucial for interpreting the data.

Figures 3B to 3D show the mean estimates for the two Kalman gains, *K*_*1*_ and *K*_*2*_, along with the general bias. Parameter recovery revealed that *K*_*1*_ and *K*_*2*_ in the realistic range can be well-recovered (Supplement 3). A volatility-by-group mixed-design ANOVA on *K*_*1*_ revealed a significant volatility effect, *F*_1,62_ = 10.61, *p* = .002, η_p_^2^ = 0.146. *K*_*1*_ was higher in the high-volatility (68.2%) than in the low-volatility (60.5%) session, indicating greater reliance on sensory input over prior knowledge in unpredictable environments. There were no main or interaction effects involving group, *F*s < 2.62, *p*s > .11, η_p_^2^s < 0.04.

For *K*_*2*_, the ANOVA showed a significant group difference, *F*_1,62_ = 4.23, *p* = .04, η_p_^2^ = 0.146: on average, individuals with ASD exhibited a higher *K*_*2*_ (0.395) than their TD counterparts (0.297), indicative of a stronger tendency of the former to revise their priors. Volatility also significantly affected *K*_*2*_, *F*_1,62_ = 11.75, *p* = .001, η_p_^2^ = 0.159: participants were more inclined to update their priors (i.e., less reliable prediction) in the high- (0.42) versus low-volatility (0.27) environment. However, the group × volatility interaction was nonsignificant, *F*_1,62_ = 1.21, *p* = .276, η_p_^2^ = 0.019.

Another mixed-design ANOVA revealed volatility as the only significant factor affecting the general bias, *F*_1,62_ = 11.83, *p* = .001, η_p_^2^ = 0.16, corroborating earlier reported behavioral results. No main or interaction effects involving group were observed, *F*s < 0.794, *p*s > .37, η_p_^2^s < 0.013).

In summary, while volatility impacted all three parameters examined, a differential effect of ASD versus TD group was observed only in the Kalman gain *K*_*2*_, which is closely related to the sequential bias.

### First Versus Second Half: Impact of Updating Parameters on the Central-Tendency Bias

**Figure 4:**
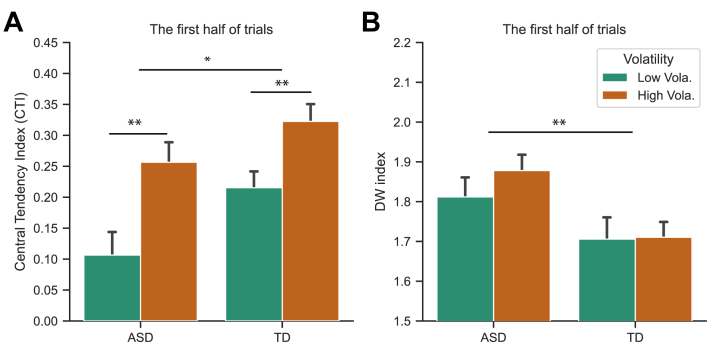
**(A)** Average central-tendency indices (CTIs) and associated standard errors for the autism spectrum disorder (ASD) and typically developing (TD) groups (each group *n* = 29), separately for the high- and low-volatility sessions. A CTI of 0 implies a lack of a central-tendency effect, whereas a CTI of 1 indicates a pronounced central-tendency effect. **(B)** Average Durbin-Watson (DW) autocorrelation indices and associated standard errors, separately for the high- and low-volatility sessions, for both the ASD and TD groups. A DW value of 2.0 signifies the absence of autocorrelation, while a DW value <2 indicates a positive autocorrelation. Error bars denote 1 standard error. ∗*p* < .05, ∗∗*p* < .01.

Higher *K*_*2*_ in ASD causes more fluctuations in updating the mean prior, leading to heavy reliance on sensory inputs, which might show differential central-tendency biases during initial stages. To further validate this, we analyzed the first half of trials in each session. Given that we only applied the first half of the trials (limiting data quality), we excluded the 3 outliers and their counterparts. Figure 4A shows mean CTIs from these initial trials. A mixed-design ANOVA revealed significant main effects of group (*F*_1,56_ = 6.236, *p* = .015, η_p_^2^ = 0.1) and volatility (*F*_1,56_ = 24.74, *p* < .001, η_p_^2^ = 0.31), which is in contrast to the nonsignificant main effect of group when tested across all trials, even when excluding the outlier individuals. The CTI was significantly smaller for the ASD group than for the TD group in these initial trials (while being comparable across all trials), indicating slower adaptation in autistic individuals. Similar to the analysis of all trials, the DW values were significantly higher for the ASD group than the TD group already during the first half of trials, *F*_1,56_ = 7.2, *p* = .01, η_p_^2^ = 0.114 (Figure 4B).

Next, we re-estimated the Kalman gains *K*_*1*_ and *K*_*2*_ based on the first half of trials in each session and compared them with the parameters obtained for the entire set of trials. Statistical tests on *K*_*1*_ and *K*_*2*_ revealed similar results as the entire session we reported earlier: *K*_*1*_ only differed significantly between volatility sessions (volatility: *F*_1,56_ = 21.89, *p* < .001, η_p_^2^ = 0.281; others: *F*s < 1.91; *p*s > .17), while *K*_*2*_ differed in both factors: group (*F*_1,56_ = 4.51, *p* = .038, η_p_^2^ = 0.075) and volatility (*F*_1,56_ = 11.18, *p* < .001, η_p_^2^ = 0.167), but not their interaction (*F*_1,56_ = 0.02, *p* = .89, η_p_^2^ < 0.001). This suggests that Kalman gains may reflect long-term steady-state updating rates. In fact, *K*_*1*_ and *K*_*2*_ remained relatively stable for the first session and the entire session (mean differences 0.006–0.058 for *K*_*1*_, and 0.007–0.022 for *K*_*2*_). A significant change was only observed in *K*_*1*_ between two volatility sessions (*F*_1,56_ = 4.31, *p* = .043, η_p_^2^ = 0.071). Specifically, there was an increase of 0.032 in *K*_*1*_ in the high-volatility condition and a decrease of 0.013 in the low-volatility condition, a net difference of 0.045 (mean *K*_*1*_: 0.643). The change was comparable between the 2 groups, *F*_1,56_ = 2.23, *p* = .141, η_p_^2^ = 0.038, indicating that both groups adjusted their weighting similarly.

Thus, the process of short-term belief updating remained relatively stable over the course of all trials, with the ASD group exhibiting a significantly higher Kalman gain *K*_*2*_ than the TD group. This difference in *K*_*2*_ influenced the rate at which long-term beliefs are formed, which in turn affected the central tendency effect. A significant difference in the central tendency effect between the 2 groups was evident in the first half of the trials, though it diminished across the entire session.

Finally, we examined potential relationships between the estimated parameters and symptom severity (Supplement 5). We only found a negative correlation between the SQ and *K*_*2*_ for both groups: individuals with higher SQ (that is, systematic thinking) scores tended to exhibit lower updating weights *K*_*2*_. However, *K*_*2*_ was significantly higher for individuals with ASD than for their TD counterparts.

## Discussion

The nature of differences in predictive processing between individuals with ASD and their TD peers remains debated (4, 5, 6, 7, 8, 9, 10, 11, 12,40, 41, 42, 43). Some studies have reported general impaired predictive processes in ASD (4, 5, 6,14), while others have found that predictive processing remains intact (11,42,43), although with atypical learning dynamics (23,24,43). We hypothesized that these discrepancies may stem from how studies conceived of the updating of prior beliefs, perceptual estimates from integration of prior knowledge and sensory input, and the time scales examined.

Using the two-state Bayesian model (29) in a duration-reproduction task under high- and low-volatility conditions, we distinguished how ASD and TD groups updated prior means and perceptual estimates. Both groups demonstrated similar central tendency effects after the entire session, consistent with recent findings (40), and both responded to changes in environmental uncertainty, showing a more pronounced central tendency under high- relative to low-volatility conditions. However, rather than overestimating or underestimating volatility per se [as suggested by the study using the hierarchical Gaussian filter model (10)], autistic participants formed appropriate priors in response to different volatile environments across the session.

Crucially, our model dissociated two distinct mechanisms via separate Kalman gains. The first gain (*K*_*1*_) weights the sensory input for immediate perceptual estimation, and both groups showed similar reliance on sensory information in this regard. The second gain (*K*_*2*_) governs the iterative updating of the prior mean based on recent experiences. Here, individuals with ASD relied more heavily on sensory input—reflected in a higher *K*_*2*_—suggesting that their internal prior distributions are updated more dynamically on a trial-by-trial basis. In other words, while the overall environmental statistics (*K*_*1*_) were accurately captured over the session, the ASD group treated successive temporal events as less interconnected, leading to more rapid adjustments of interim priors (*K*_*2*_). This differential updating process may explain differences in the temporal dynamics observed in our task. Early in the session, autistic participants exhibited a smaller central-tendency effect than TD participants, consistent with previous reports (4,14). However, these differences diminished over time, and both groups converged on a similar representation of the distribution.

In short, our two-state Bayesian model (29) distinguishes between immediate perceptual adjustments (*K*_*1*_) and slower, iterative updates to the prior (*K*_*2*_). The increased weighting of sensory input in the updating of priors among individuals with ASD does not imply a misestimation of environmental volatility but rather indicates that they are perceiving temporal events as less interconnected than their TD counterparts. These results support the idea that individuals with ASD can accurately gauge environmental statistics, although their intertrial updating processes differ from those of TD control participants (19).

This atypicality in intertrial prior updating is partially consistent with recent suggestions that ASD is characterized by slower adaptation (23,24). For example, Lieder *et al.* (23) found that in a tone-discrimination task, individuals with ASD weighted the immediately preceding stimulus less than control participants despite similar long-term weighting. However, our study highlights a different pattern: the groups did not differ in integrating past information into perceptual estimates but in updating internal prior beliefs. A general linear model analysis (Supplement 6) further confirmed this distinction. While the analysis showed differences in the weighting of preceding durations (i.e., the sequential dependence) between high- and low-volatility sequences, it found no group differences between ASD and TD participants, suggesting that the observed differences are specific to prior updates rather than sensory integration.

The distinctive updating of prior beliefs in ASD is not inherently a slower process but is marked by heavier reliance on sensory inputs, which can slow adaptation over time. In uncertain environments, this sensory-driven approach is advantageous, enabling quicker responses to immediate stimuli and a less pronounced central-tendency bias early in a session. However, over longer periods, such as an entire session, this reliance results in slower behavioral adaptation, potentially contributing to the behavior rigidity observed in individuals with ASD.

Thus, our findings may help explain some mixed findings on predictive processing in ASD (15), which often depend on the rate of adaptation and the timescales used in studies. For example, some studies have indicated intact sensitivity to interval timing in ASD (40,44,45), while others have reported reduced sensitivity, especially with certain intervals or tasks (41,46, 47, 48). Notably, Karaminis *et al.* (14) found that children with ASD exhibited a stronger central tendency and poorer temporal resolution in duration reproduction than their TD counterparts. However, their central tendency was weaker than predicted by Bayesian integration, implying poorer priors in the ASD group. Notably, their study involved only 77 trials per whole session for child participants, fewer than the first half of trials in our sessions (i.e., 125 trials). Accordingly, their findings may reflect an initial low weight of prior beliefs during sensory-prior integration, consistent with our observations in early session trials. One prediction deriving from our findings is that extended testing may allow children with ASD to develop priors similar to those of their TD counterparts.

It is worth noting that in the ASD group, 3 individuals of 32 exhibited markedly different behavior, showing strikingly similar response patterns among themselves (see Supplement 1). In the low-volatility session, they reproduced durations proportional to the target stimuli, although with some general over- or underestimations. However, in the high-volatility environment, they consistently reproduced the same duration across all intervals, ignoring sensory inputs (with *K*_*1*_ and *K*_*2*_ values at 0.07 and 0.04, respectively) and relying solely on an overly strong internal prior. Their normal performance in the low-volatility sequence rules out a misunderstanding of the instructions. Interestingly, during the debriefing, these 3 individuals reported no awareness of the difference in the volatility regimen between the 2 sessions, suggesting that their performance was influenced implicitly. We hypothesize that when experiencing a highly volatile stimulus sequence, they treated large, uncorrelated stimulus changes as noise rather than signal, effectively discounting sensory inputs. That is, they seem to change their model between high- and low-volatility environments concerning the likelihood distribution, the assumption about what is noise and what is signal. It should be noted that even when we included these outliers in our analysis, the critical results of the 2-state model remained consistent.

### Conclusions

In summary, our findings offer a new perspective on predictive-processing dynamics in ASD and clarify apparent inconsistencies in the literature. Individuals with ASD use prior knowledge similarly to control participants but place greater emphasis on sensory information when updating interim priors, distinct from updating percepts. This distinctive process may lead to slower adaptation of perceptual decision making over time, which, depending on the environmental volatility, may not necessarily be a disadvantage. Discrepancies in the extant literature may stem from different studies focusing on different timescales, overlooking the dynamics of these two distinct updating processes.

Our results indicate that individuals with ASD adapt appropriately to both volatile and stable environments but exhibit unique intertrial dynamics. Specifically, the two-state iterative updating model highlights their focus on temporal discontinuity and overreliance on sensory input during moment-to-moment prior updating, both of which are characteristic of predictive processing in ASD.

## Supplementary Material

Supplement Methods, Results, Figures S1–S7, and Tables S1–S2

Key Resources Table
