# Supplementary material for: Predictive Processing in Autism Spectrum Disorder: The Atypical Iterative Prior Updating Account
Source: Biol Psychiatry Glob Open Sci. 2025 Feb 17;5(3):100468. doi: 10.1016/j.bpsgos.2025.100468 (PMC11994323; doi:10.1016/j.bpsgos.2025.100468)
Supplement: Supplement Methods, Results, Figures S1–S7, and Tables S1–S2 [file mmc1.pdf]

SUPPLEMENTARY INFORMATION

Predictive Processing in Autism Spectrum Disorder: The Atypical Iterative Prior Updating Account

Shi *et al.*

Supplement 1: Descriptive characteristics of the ASD and TD groups

The groups were matched pairwise using the 'Wortschatztest'(WST, 1), a measure of crystalline intelligence. Both groups completed the Autism-Spectrum Quotient (AQ, 2), Empathy Quotient (EQ, 3), Systemizing Quotient (SQ, 4), and Beck's Depression Inventory (BDI, 5). The mean scores and paired *t*-tests are shown in Table S1. As can be seen, the AQ, EQ, SQ, and BDI scores were significantly different between the two groups.

Table S1. *Descriptive characteristics (Means and SDs) for ASD and TD group.*

Measures	*ASD (n=32)*	*TD (n=32)*	*Group comparison*
Age	32.03 (12.3)	31.6 (13.6)	$t_{31} = 0.13$, $p = .898$
IQ score	107.4 (9.7)	109.1 (13.4)	$t_{31} = 0.573$, $p = .28$
Autism-Spectrum Quotient score	36.7 (7.4)	15.8 (6.0)	$t_{31} = 18.06$, $p < .001^{**}$
Empathy Quotient score	26.97 (12.32)	50.38 (14.46)	$t_{31} = -7.85$, $p < .001^{**}$
Systemizing Quotient score	35.25 (14.93)	26.63 (10.1)	$t_{31} = 3.05$, $p < .01^{**}$
Beck's Depression Inventory score	10.69 (8.37)	5.25 (7.46)	$t_{31} = 2.49$, $p = .018^{*}$

Note: ** denotes p < .05 and ** p < .001.

Supplement 2. Duration reproduction in the high- (vs. low-) volatility session by three 'outlier' individuals in the ASD group

In the high-volatility session, three participants from the ASD group exhibited a markedly different performance compared to the others in the group (see Figure 3 Boxplots). To better visualize their reproduction performance, we replot their reproduced durations as a function of the physical duration. Figure S1 illustrates their performance in the low- and high-volatility sessions. As can be seen, the reproduced durations were similar across the range of durations in the high-volatility session (orange, 'flat'), even though these individuals performed similarly to their peers in the low-volatility session. For these three outliers, the central-tendency indices (1 – regression coefficient) were extremely high (0.904, 0.997, and 0.907, respectively) in the

high-volatility session. This suggests that these individuals relied heavily on their prior instead of the sensory inputs for their duration judgments, rendering a remarkable level of rigidity in their responses.

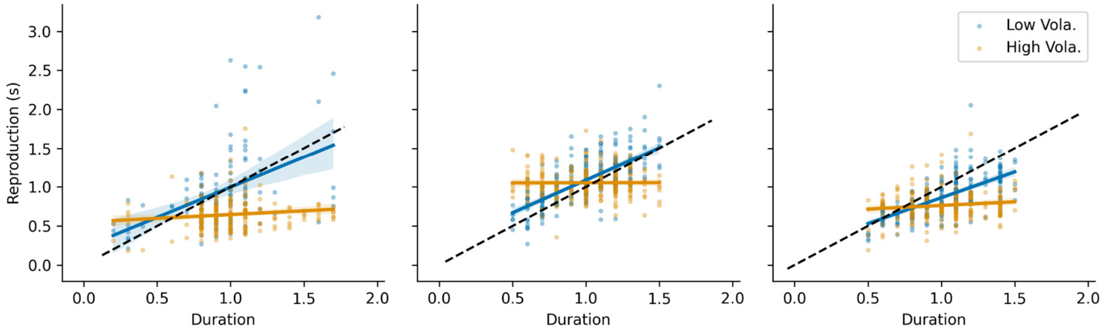

Figure S1. Duration reproduction by three 'extreme' ASD individuals, separately for the high- (orange) and low-volatility (blue) sessions. The diagonal dashed line denotes veridical reproduction.

Supplement 3. The two-state model

The Model

The generative process describes how the two states of the model, the stimulus duration (x_i) and the mean of the stimulus distribution (m_i) on the current trial i, evolve over time:

$$x_i = m_{i-1} + \epsilon_x, \quad (1)$$

$$m_i = m_{i-1} + \epsilon_m, \quad (2)$$

where ϵ_x and ϵ_m are normally distributed noise with variances v and q, respectively. Thus, it is assumed that the mean of stimulus distribution m_i changes by a random amount each trial, and that the actual stimulus x_i is drawn from that distribution. The actual sensory measurement z_i is the stimulus (x_i), corrupted by the sensory noise $\eta \sim N(0, r)$:

$$z_i = x_i + \eta. \quad (3)$$

The estimate of the two states can be written as:

$$\hat{x}_i = \hat{m}_{i-1} + K_1(z_i - \hat{m}_{i-1}) + c \quad (4)$$

$$\hat{m}_i = \hat{m}_{i-1} + K_2(z_i - \hat{m}_{i-1}) \quad (5)$$

where K_1 and K_2 are the Kalman gains to determine the estimates of the current estimate $\hat{x}_i$, and the mean of the stimulus distribution $\hat{m}_i$, respectively, and c is a constant bias. The Kalman

gain K_i is essentially a weighting factor determining the weight assigned to the new prediction error $(z_i - \hat{m}_{i-1})$. If the Kalman gain is high, the system places more trust on the new sensory information; if it is low, it places greater trust on the prediction (prior).

To ensure that the two-state model performs well for the behavioral data, we fitted the model with three parameters: the variance ratio *v/r*, the variance ratio *q/r*, and the constant bias *c*. The specifics of the fitting procedure can be found in our earlier work (Glasauer & Shi, 2022).

Predictions from the two-state model

The Kalman gains of the model are closely related to the central tendency effect and sequential bias. As we can see from the analytical proof below, K_1 determines the central tendency bias, while K_1 and K_2 together determine the sequential bias in a steady state.

From Eqs (4) and (5), we can rewrite

$$\hat{x}_i = K_1 z_i + (1 - K_1)(K_2 z_{i-1} + (1 - K_2)\hat{m}_{i-2}) \quad (6)$$

Considering $\hat{x}_{i-1} = K_1 z_{i-1} + (1 - K_1)\hat{m}_{i-2}$, we can obtain the following relation:

$$\hat{x}_i = K_1 z_i + (1 - K_2)\hat{x}_{i-1} + (K_2 - K_1) z_{i-1}, \quad (7)$$

which indicates the autoregressive nature of the two-state model. K_1 indicates the central tendency bias. The factor $(K_2 - K_1)$ in Eq. (7) does not correspond to the sequential bias, because $\hat{x}_{i-1}$ and z_{i-1} are not independent. But we can reformulate the model using

$$\hat{x}_{i-1} = K_1 z_{i-1} + (1 - K_2)\hat{x}_{i-2} + (K_2 - K_1) z_{i-2},$$

which yields

$$y_i = K_2(1 - K_1) z_{i-1} + (1 - K_2)^2 y_{i-2} + (1 - K_2)(K_2 - K_1) z_{i-2} + K_1 z_i \quad (8)$$

Given that the sequential dependence is quantified as current error depending on previous input ($\hat{x}_i - z_i = a + b \cdot z_{i-1}$), the coefficient $K_2(1 - K_1)$ for z_{i-1} in Eq. (8) is independent of all others and constitutes the sequential dependence if the stimuli are IID distributed, thus representing the sequential bias. For $K_2 = 0$ the sequential dependence is zero.

To further demonstrate the relationships between Kalman gains (K_1 and K_2) and central tendency and sequential bias, we simulated the model's performance on reproduction error for stimulus durations. The model imposes the constraint $0 \leq K_1 - K_2 < 1$, meaning K_2 is always smaller or equal to K_1, since additionally both gains are bounded between 0 and 1. Consequently, the sequential dependence reaches its upper bound when $K_1 = K_2$ with $K_2 - K_2^2$. We used the steady-state version of the model to directly adjust the Kalman gains, which

are derived from the fitted parameters of the model: the variance ratios q1/r and q2/r (see Glasauer & Shi 2021).

As shown in Figure S2, central tendency depends on K_1 rather K_2. Furthermore, the central tendency effect is smaller during the low-volatility session compared to the high-volatility session.

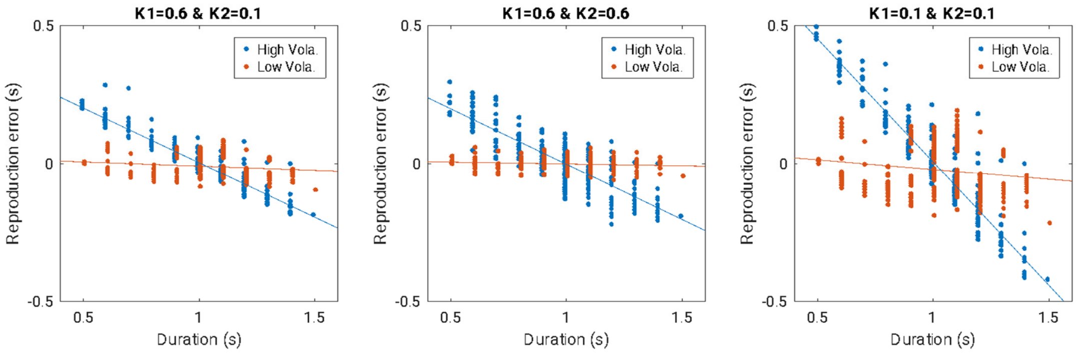

Figure S2: Model simulations demonstrating the effect of changes in Kalman gains K_1 and K_2 on the reproduction error plotted over *current* stimulus duration. Left: Realistic gains K_1=0.6 and K_2=0.1 show a strong central tendency for the high volatility condition, while little systematic error for the low volatility condition. Middle: Increasing K_2 to 0.6 has no clearly visible effect. Right: In contrast, when K_1 is decreased to 0.1, the central tendency in the high volatility condition becomes much more pronounced.

In contrast, the sequential bias (the relationship between the current error and the previous duration) depends on both gains, as indicated by the analytical result $[(K_2(1-K_1)]$. Figure S3 shows that under the high volatility condition, sequential bias increases as K_2 increases. Additionally, when both gains are small, the sequential dependence may becomes more pronounced.

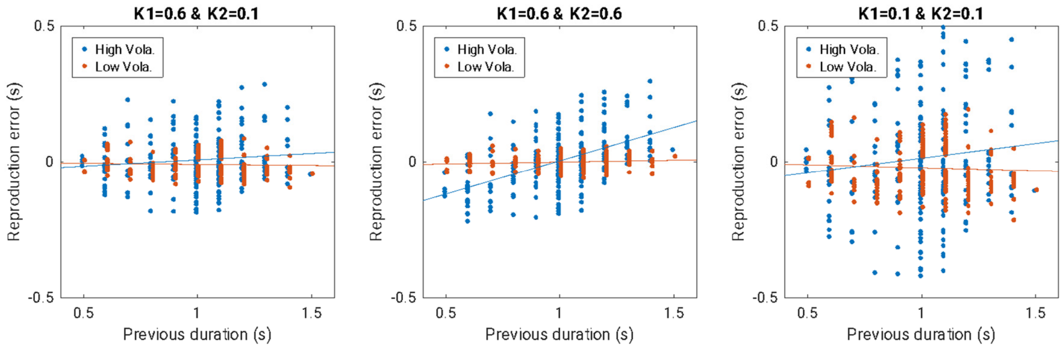

Figure S3: Model simulations demonstrating the effect of changes in Kalman gains K_1 and K_2 on the reproduction error plotted over *previous* stimulus duration. Left: a small positive

sequential dependence for the high volatility condition and a very small negative one for the low volatility condition can be observed. Middle: Increasing K_2 to 0.6 causes a clear increase in sequential dependence. Right: when both K_1 and K_2 are small, the sequential dependence in the high volatility condition becomes more pronounced than in the left case where K_1 is large.

Parameter recovery

The two-state model is a non-linear Bayesian framework of magnitude perception. It takes the sensory measurement of a stimulus magnitude as input and produces the perceived magnitude as output. Although the model incorporates uncertainties of sensory measurement and stimulus generation using probability densities, it operates deterministically. This means that when applied repeatedly to the same series of inputs, it will always generate the same series of outputs. Experimentally, however, we only have access to the stimulus magnitudes, not the sensory measurements received by observers, and we have only access to the reproduced duration, but not the perceived duration. For model fitting, we therefore approximate the sensory input with the stimulus magnitude and the perceived magnitude with reproduced magnitude responses. Consequently, for a given set of parameters and a specific stimulus time series, the model's responses are always identical.

To evaluate parameter recovery, we first fitted the model to each participant's data. Next, we ran the model with the fitted parameters and original stimulus time series to generate simulated responses. We then re-fitted the model to the simulated responses and compared the recovered parameters to the original ones used in the simulation.

As shown in Fig. S4, parameter recovery was successful for all tested parameter sets using stimulus duration as input. This result is expected, since the model itself is deterministic, the parameters are non-redundant, and thus the simulated responses constitute the optimum for the given parameter set. When expressing the model parameters as Kalman gains K_1 and K_2 and bias, the maximum error for recovery was 0.00066 for K_1, 0.0017 for K_2 , and 0.00002 for the bias term, which probably results from numerical errors.

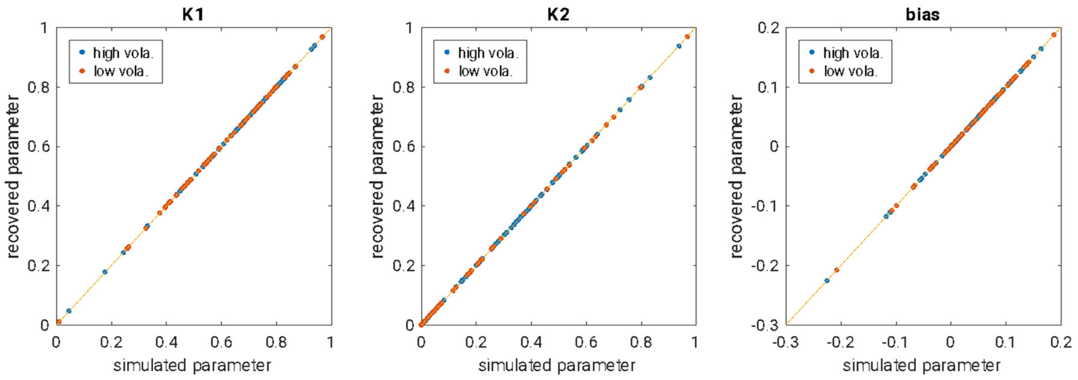

Figure S4: Recovered parameters plotted over true simulated parameters for simulation without noise. Note that the fitted first two parameters model of the model are the variance ratios q_{11}/r and q_{22}/r, which, however, can vary substantially in size. We, therefore, plotted the steady-state Kalman gains instead, which correspond to weights and, therefore, are bounded between 0 and 1. Parameter recovery was basically perfect, as indicated by all points lying on the diagonal. Blue and red dots indicate conditions with high and low volatility, and each dot corresponds to a parameter determined by one of the participants.

As mentioned above, so far we used stimulus magnitude as a proxy for sensory measurement. However, to conduct a more realistic parameter recovery test, we considered that the actual sensory measurement on which the modeled perceptual process would need to operate is the stimulus magnitude contaminated by sensory noise. We therefore ran a second series where we also simulated the sensory noise. To do so, we estimated the individual noise level of each participant as error variance and used this to model the individual strength of sensory noise. Note that this approach likely overestimates sensory noise because it attributes all variability to the sensory measurement, while in reality, a portion of the variability likely arises from other noise sources, such as motor execution noise. Sensory noise was simulated as Gaussian additive noise.

Using these noise parameters, we simulated the model as before, applying each participant's parameters but using 1000 noisy stimulus time series as inputs. As evident from Fig. S5, the recovered parameters exhibited considerable variability. However, on average, recovery remained accurate for K_1, while showing some issues for K_2 near zero and above 0.8, and revealed a small but consistent underestimation of the bias parameter.

Paired t-tests comparing the true and average recovered parameter values confirmed these observations. For K_1 and K_2, no significant differences were found between true and recovered parameters (paired t-test $p>0.05$). Recovery was slightly better, in terms of mean

absolute error, for both K_1 and K_2 in the high-volatility condition compared to the low-volatility condition (mean error for k1: 0.0069 vs. 0.0207, for k2: 0.0332 vs. 0.0612). In contrast, the bias term showed a highly significant difference (t-test $p<0.0001$), with mean errors of 0.016 for the high-volatility condition and 0.011 for the low-volatility condition. It should be noted that these results are highly dependent on how sensory noise is estimated and approximated.

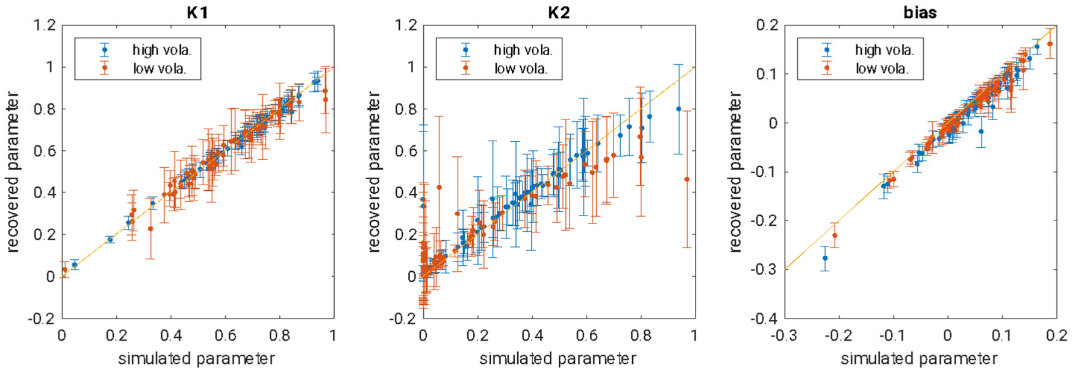

Figure S5: Recovered parameters plotted over simulated parameters with simulated sensory noise. True parameters as in Figure 1. Error bars indicate standard deviation of recovered parameters. Each parameter set was simulated 1000 times.

Supplement 4

We compared our two-state model to the internal reference model (IRM, 6,7), which assumes that the internal reference $\boldsymbol{I_n}$ on trial n is a weighted sum of the internal reference $\boldsymbol{I_{n-1}}$ from the previous trial and the internal representation of the current stimulus duration $\boldsymbol{D_n}$:

$$I_n = g \cdot I_{n-1} + (1-g) \cdot D_n + b, \qquad (9)$$

where the integration weight g may vary between groups and volatility conditions, and b is a general bias (not included in the original model). During reproduction, participants compare the currently elapsed duration until it matches the current internal reference (7). The IRM has two parameters, fewer parameters than the three-parameter two-state model. Given that the IRM is a linear model, we estimated its parameters using linear regression. To calculate the model error, we iteratively simulated the model using the fitted parameters, setting the first reproduction as the initial value for the internal reference (alternatively, one could also use the first stimulus).

Surprisingly, the integration weight *g* of the IRM was neither significant difference between high and low volatility sessions, F(1,62) = 1.10, p = 0.30 (numerically higher g in the high than the low volatility session), nor between the ASD and TD groups, F(1,62) = 0.87, p=0.35. And their interaction was also not significant (Figure S6). These results indicated that IRM may not be sensitive to the volatility manipulation implemented in the study. Further model comparison based on AIC indicated that the two-state model provided a better overall fit than the IRM. An ANOVA on AIC confirmed that AIC values were significantly lower for the two-state model than for the IRM in both two volatility sessions (F(1,62)=27.0, $p < .001$).

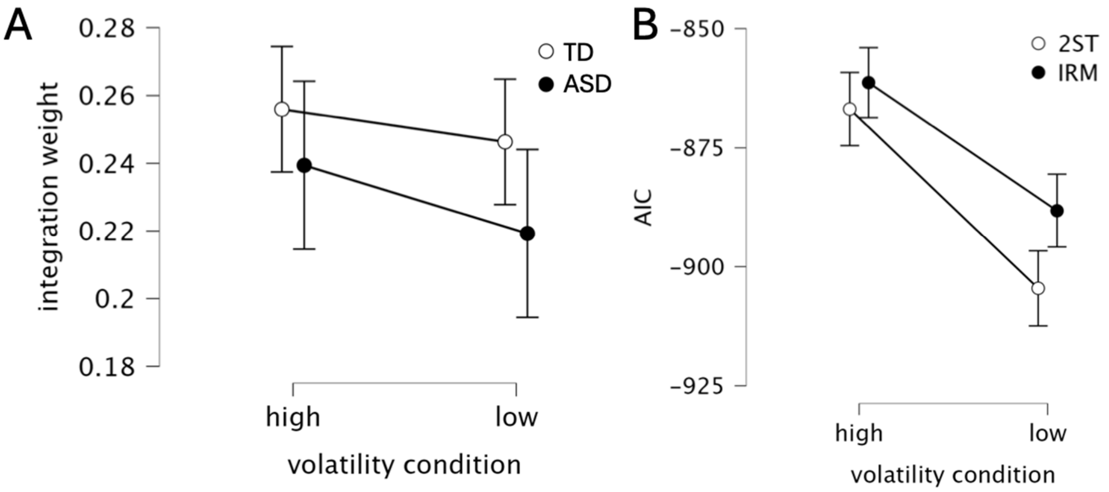

Figure S6. A: Integration weight parameter *g* for the internal reference model (IRM). Neither "group" (TD and ASD) nor condition (high vs. low volatility) showed a significant effect. B: Model comparison between the two-state model (2ST) and the internal reference model (IRM). The two-state model had significantly lower AICs compared to the IRM independently of volatility condition. Error bars denote standard error.

Supplement 5. Correlations between the updating weight and symptom severity

We examined for potential relationships between the estimated K_2 parameter and symptom severity, measured by the Autism-Spectrum Quotient (AQ), Empathy Quotient (EQ), Systemizing Quotient (SQ), Beck's Depression Inventory (BDI), and Empathising-systemising (E-S) scores. In addition, we considered potential contributions from the two experimental factors *Group* and *Volatility*. We found a strong negative correlation between the K_2 and SQ scores ($p = .003$), and a strong positive correlation between the K_2 and E-S ($p = .016$) scores. The estimated linear models were:

$$K_2 = 0.66 - 0.155\ G_{TD} - 0.158\ V_{low} - 0.005\ SQ, \quad (10)$$

$$K_2 = 0.496 - 0.195\ G_{TD} - 0.158\ V_{low} - 0.003\ ES, \quad (11)$$

where G_{TD} is a dummy variable for Group (1 for the TD group, 0 for the ASD group) and V_{low} for the Volatility (1 for the low-volatility session, and 0 for the high-volatility session). The intercepts and coefficients for the dummy variables were all significant ($p < .001$). K_2 was significantly higher for the ASD group compared to the TD group (15.5% and 19.5%, respectively, in the above two estimated models). Figure S2 shows the correlation trends for both scores. Given that the E-S scores were derived from the difference between the Systemizing Quotient (SQ) and Empathy Quotient (EQ) scores, SQ is likely the main driving factor of the correlation.

The correlation suggests that the updating weight K_2 is sensitive to the individual preference and aptitude for systemizing: the higher the systemizing preference, the smaller the updating weight of the sensory data in the formation of the prior beliefs.

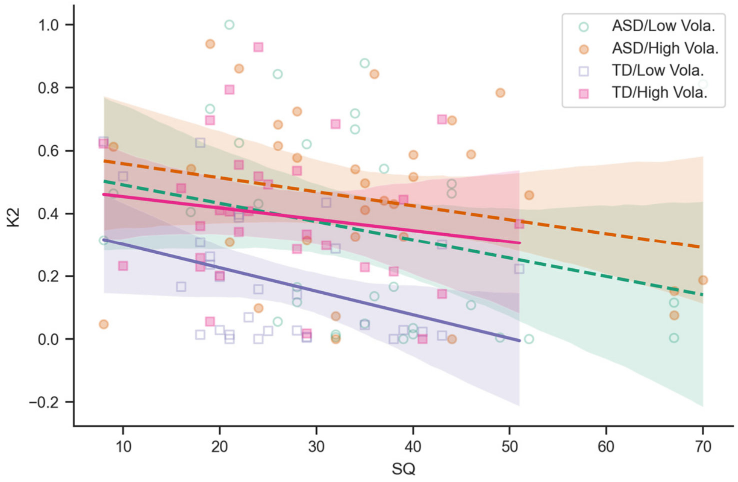

Figure S7. Negative correlations between the K_2 and Systemizing-Quotient (SQ) scores, separately for the ASD/Low-volatility (green circles, dashed line) and ASD/High-volatility (red circles, dashed line), TD/Low-volatility (light blue squares, solid blue line), TD/High-volatility conditions (pink squares, pink solid line).

Supplement 6. A general linear model (GLM) considering the preceding trial

To examine whether there were any differences in how the two groups incorporated short-term trial history, we conducted a general linear model (GLM) that factored in both the

current and the immediately preceding durations to predict the current reproduction errors. Given that in the low-volatility session, the current and previous durations were not fully independent, we used the duration difference of the current from the preceding trial ($\Delta D = D_n - D_{n-1}$) in our regression model. Additionally, to mitigate the impact of extreme performance, we removed the three 'outlier' individuals and their matched counterparts from the analysis. Table S2 displays the average regression coefficients and their associated standard errors.

We then performed separate mixed-design ANOVAs for each regression coefficient, with *Group* and *Volatility* as factors. These analyses failed to reveal any significant *Group* or *Volatility* effects on the intercept. The coefficients for the Current Duration D_n differed significantly between the high- and low-volatility sessions, $F(1,56) = 24.57, p < .001$, η_p^2=.305. However, the main effect of Group and the Group x Volatility interaction failed to reach significance, Fs < 2.218, ps > .14, η_p^2s< .038. Similarly, the coefficients for the deviation ΔD differed significantly between the high- and low-volatility sessions, F(1,56) = 54.49, p < .001, η_p^2=.489; but again, there were no (main or interaction) effects involving *Group*, Fs < 3.34, ps > .073, η_p^2s < .056. We take this to indicate that the 'history' impact of the preceding Duration was similar for both groups.

Table S2. Average regression coefficients and their associated standard errors (in parentheses) for the ASD and TD groups, separately for the high- and low-volatility sessions.

Group	Volatility	Intercept	Current Duration (D_n)	$D_n - D_{n-1}$
ASD	High	0.032 (0.015)	-0.166 (0.026)	-0.104 (0.016)
	Low	0.042 (0.015)	-0.092 (0.030)	-0.281 (0.041)
TD	High	0.029 (0.011)	-0.224 (0.023)	-0.123 (0.012)
	Low	0.038 (0.013)	-0.086 (0.021)	-0.394 (0.049)

References

1. Schmidt KH, Metzler P (1992): WST-Wortschatztest. *Göttingen: Beltz Test*.

2. Baron-Cohen S, Wheelwright S, Skinner R, Martin J, Clubley E (2001): The Autism-Spectrum Quotient (AQ): Evidence from Asperger Syndrome/High-Functioning Autism, Males and Females, Scientists and Mathematicians. *J Autism Dev Disord* 31: 5–17.

3. Baron-Cohen S, Wheelwright S (2004): The empathy quotient: an investigation of adults with Asperger syndrome or high functioning autism, and normal sex differences. *J Autism Dev Disord* 34: 163–175.

4. Baron-Cohen S, Richler J, Bisarya D, Gurunathan N, Wheelwright S (2003): The systemizing quotient: an investigation of adults with Asperger syndrome or high-functioning autism, and normal sex differences. *Philos Trans R Soc Lond B Biol Sci* 358: 361–374.

5. Beck AT, Steer RA, Brown G (2011): Beck Depression Inventory–II. *PsycTESTS Dataset*. https://doi.org/10.1037/t00742-000

6. Dyjas O, Bausenhart KM, Ulrich R (2012): Trial-by-trial updating of an internal reference in discrimination tasks: evidence from effects of stimulus order and trial sequence. *Atten Percept Psychophys* 74: 1819–1841.

7. Bausenhart KM, Dyjas O, Ulrich R (2014): Temporal reproductions are influenced by an internal reference: explaining the Vierordt effect. *Acta Psychol* 147: 60–67.
